# Targeted Modification of Mammalian DNA by a Novel Type V Cas12a Endonuclease from *Ruminococcus bromii*

**DOI:** 10.3390/ijms23169289

**Published:** 2022-08-18

**Authors:** Ruslan Vasilev, Natalia Gunitseva, Regina Shebanova, Aleksei Korzhenkov, Anna Vlaskina, Marta Evteeva, Irina Polushkina, Natalia Nikitchina, Stepan Toshchakov, Piotr Kamenski, Maxim Patrushev, Ilya Mazunin

**Affiliations:** 1 Kurchatov Genomics Center, National Research Center “Kurchatov Institute”, 123098 Moscow, Russia; 2 Faculty of Biology, Lomonosov Moscow State University, 119991 Moscow, Russia; 3 Center of Life Sciences, Skolkovo Institute of Science and Technology, 143026 Moscow, Russia; 4 UMR7156–Molecular Genetics, Genomics, Microbiology, University of Strasbourg and Centre National de la Recherche Scientifique (CNRS), 67000 Strasbourg, France; 5 Medical Genomics LLC, 119192 Moscow, Russia

**Keywords:** Cas endonuclease, CRISPR, genome editing, mammalian cells, site-directed mutagenesis

## Abstract

Type V Cas12a nucleases are DNA editors working in a wide temperature range and using expanded protospacer-adjacent motifs (PAMs). Though they are widely used, there is still a demand for discovering new ones. Here, we demonstrate a novel ortholog from *Ruminococcus bromii* sp. entitled RbCas12a, which is able to efficiently cleave target DNA templates, using the particularly high accessibility of PAM 5′-YYN and a relatively wide temperature range from 20 °C to 42 °C. In comparison to *Acidaminococcus* sp. (AsCas12a) nuclease, RbCas12a is capable of processing DNA more efficiently, and can be active upon being charged by spacer-only RNA at lower concentrations in vitro. We show that the human-optimized RbCas12a nuclease is also active in mammalian cells, and can be applied for efficient deletion incorporation into the human genome. Given the advantageous properties of RbCas12a, this enzyme shows potential for clinical and biotechnological applications within the field of genome editing.

## 1. Introduction

CRISPR/Cas systems are hosted in archaeal and bacterial genomes, at times within their mobile elements, and are generally accepted as a type of “immune system” for protection against nucleic acid invaders. These systems contain short fragments of invader DNA, that is, spacers surrounded by repetitive nucleotide sequences called direct repeats. Depending on the CRISPR subtype variant, *cas* genes can be located downstream or upstream from the repeats at the same locus and encode spacer integration elements, including the genes encoding the effector complex, *cas1*, *cas2*, and occasionally *cas4*. The effector module involved in target DNA cleavage consists of several independent proteins, in the case of the Class 1 system, or a single multidomain protein, in the case of the Class 2 system. Effectors are typically programed using one or two RNA molecules for successful target recognition. Most CRISPR/Cas systems require the presence of a protospacer-adjacent motif (PAM), which allows the complex to distinguish spacers already located at CRISPR modules from the DNA of novel invaders [1,2,3]. The first effector, Cas9 of *Streptococcus pyogenes*, was discovered in 2012, and is currently the most widely used RNA-guided DNA endonuclease in many areas of life sciences [4]. SpCas9 requires a G-rich PAM and two RNA molecules, crRNA and tracrRNA, which can be artificially combined in the format of a single-guide RNA (sgRNA). The assembled nucleoprotein complex in the presence of a dsDNA matrix generates RNA-guided, blunt-ended, double-strand breaks 3 nts upstream of the PAM site. During the last decade, a number of Cas9 orthologs have been discovered, characterized, and modified, providing more sensitive, efficient, and/or PAM-independent enzymes in the arsenal of researchers and commercial companies [5]. Another recently discovered ortholog, Cas12a [6], can be considered as an alternative to Cas9 due to its distinct features. Firstly, its ability to address T-rich PAMs allows for the editing of additional target motifs. Secondly, it needs to be charged with a short single RNA, which is easier to synthesize or encode for cell delivery agents. Thirdly, Cas12a induces 5′-staggered double-stranded DNA breaks in contrast to Cas9. Fourth, one of the important differences between Cas9 and Cas12a is the ability of Cas12a to process crRNA. Finally, there is an unpredictable activity of Cas12a proteins required to exert collateral activity in relation to any ssDNA molecules after dsDNA target cleavage. This property has been successfully adapted for a number of possible Cas12a-based diagnostic tools [7,8,9].

To the best of our knowledge, there have been 19 Cas12a orthologs applied for genome editing in human cells thus far [10,11,12,13,14,15,16] (Table S1). Due to the heat sensitivity of plant cells above room temperature, the search for temperature-tolerant Cas12a orthologs, as well as the development of temperature-insensitive Cas12a mutants, is of particular interest for plant genome editing. In this study, we addressed some biochemical features and the fundamental possibility of using the novel Cas12a ortholog from *Ruminococcus bromii* sp., called RbCas12a. We demonstrate that RbCas12a is an active RNA-guided DNA endonuclease that can efficiently introduce double-stranded breaks in DNA fragments with a three-nucleotide 5′-YYN PAM, where Y represents C or T. The logo 5′-YYN was proved by testing all possible combinations with an in vitro cleavage experiment. The RbCas12a was found to function well in a temperature range of 25–42 °C. We also demonstrate that RbCas12a can be used to edit genomic DNA contained in human HEK293T cells. Based on our findings, we believe that RbCas12a has the potential to become a molecular tool, providing an expanded range of target motifs in genome engineering applications.

## 2. Results

### 2.1. Characterization of CRISPR–Cas12 Subtype VA Endonuclease from Ruminococcus bromii sp.

**Figure 1:**
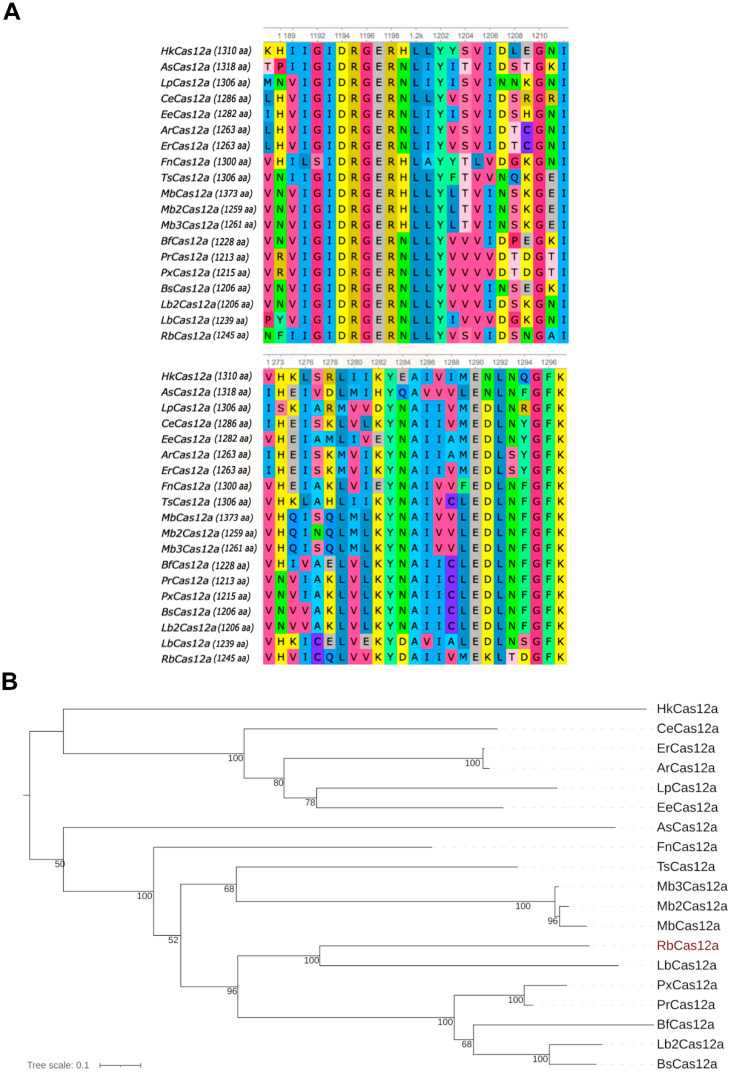
Relationship of RbCas12a to other Cas12a orthologs. (**A**) Multiple sequence alignments of RbCpf1 with common orthologs of 18 Cas12a proteins that function in human cells. Catalytic Asp1194 and Glu1290 residues (numeration shown here for total alignment) are conserved (HkCas12a, *Helcococcus kunzii* ATCC 51366; CeCas12a, *Coprococcus eutactus* sp.; ErCas12a, *Eubacterium rectale* sp.; ArCas12a, *Agathobacter rectalis* strain 2789STDY5834884; LpCas12a, *Lachnospira pectinoschiza* strain 2789STDY5834886; EeCas12a, *Lachnospira eligens* ATCC 27750; AsCas12a, *Acidaminococcus* sp. BV36L; FnCas12a, *Francisella novicida* U112; TsCas12a, *Thiomicrospira* sp. XS5; Mb3Cas12a, *Moraxella bovoculi* sp.; Mb2Cas12a, *Moraxella bovoculi* AAX08_00205; MbCas12a, *Moraxella bovoculi* 237; RbCas12a, *Ruminococcus bromii* sp.; LbCas12a, *Lachnospiraceae bacterium* ND2006; PxCas12a, *Pseudobutyrivibrio xylanivorans* strain DSM 10317; PrCas12a, *Pseudobutyrivibrio ruminis* CF1b; BfCas12a, *Butyrivibrio fibrisolvens* MD2001; Lb2Cas12a, *Lachnospiraceae bacterium* MA2020; BsCas12a, *Butyrivibrio* sp. NC3005). (**B**) Neighbor-joining tree without distance corrections.

Comparative sequence and protein architecture analysis showed that the novel nuclease from *Ruminococcus bromii* was most closely related to the CRISPR–Cas12a Type V family of enzymes, which contain a single RuvC nuclease domain that is responsible for RNA-guided DNA cleavage (Figure 1). Catalytic Asp1194 and Glu1290 amino acid residues (numeration for multiple alignment), which are conserved in all known Cas12a orthologs, were also present in RbCas12a.

**Figure 2:**
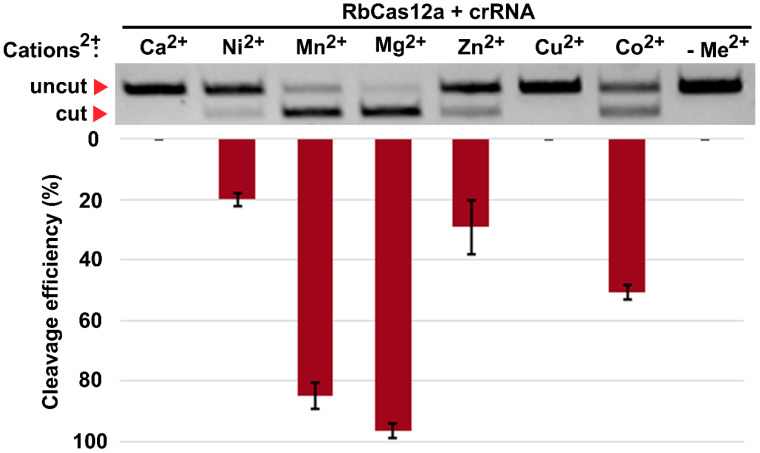
Effect of divalent cations on RbCas12a cleavage activity. Mean cleavage efficiencies and standard deviations calculated from three independent experiments of Me^2+^-dependent in vitro cleavage reactions are shown. Reactions in the same buffer but without any Me^2+^ addition served as controls.

Because of the sequence and protein architecture similarity between RbCas12a and other Cas12a orthologs, and LbCas12a in particular (Figure 1B), we chose to use 5′-TTTG PAM for initial experiments. In order to study the RbCas12a system requirements, we performed an in vitro DNA cleavage assay using the purified RbCas12a nuclease, chemically synthesized crRNAs, and PCR-derived DNA substrate. According to published data, a number of different noncustom cleavage buffers, as well as NEBuffers, for Cas12a-family nucleases, have been used. We were interested in determining which of the buffers was the most suitable for in vitro DNA cleavage by the RbCas12a nuclease. We found no differences in the tested buffers, and chose to use NEBuffer 2 in the experiments referred to below (Figure S1). The reaction products were separated by gel electrophoresis and visualized by ethidium bromide staining, and the extent of DNA substrate cleavage was quantified as described in the Materials and Methods. To further examine RbCas12a properties, we were interested in determining whether divalent cations could affect RbCas12a cleavage ability. Seven ions, including Ca^2+^, Ni^2+^, Mn^2+^, Mg^2+^, Zn^2+^, Cu^2+^, and Co^2+^ were investigated as potential cofactors of the enzyme. As shown in Figure 2, Mn^2+^ and Mg^2+^ had a comparable effect on the RbCas12a cleavage activity, with only slightly higher efficiency observed for Mg^2+^, whereas Ni^2+^ and Zn^2+^ showed low activity of the nuclease. Although RbCas12a retained the ability to cleave the target in the presence of Co^2+^ ions, only half of the DNA was found to be cleaved in three series of experiments. Ca^2+^ and Cu^2+^ drastically affected the reactions without any traces of cleavage found on the gel. As expected, the control reaction with no divalent cation addition showed the absence of endonucleolytic activity.

### 2.2. PAM Requirement of RbCas12a

**Figure 3:**
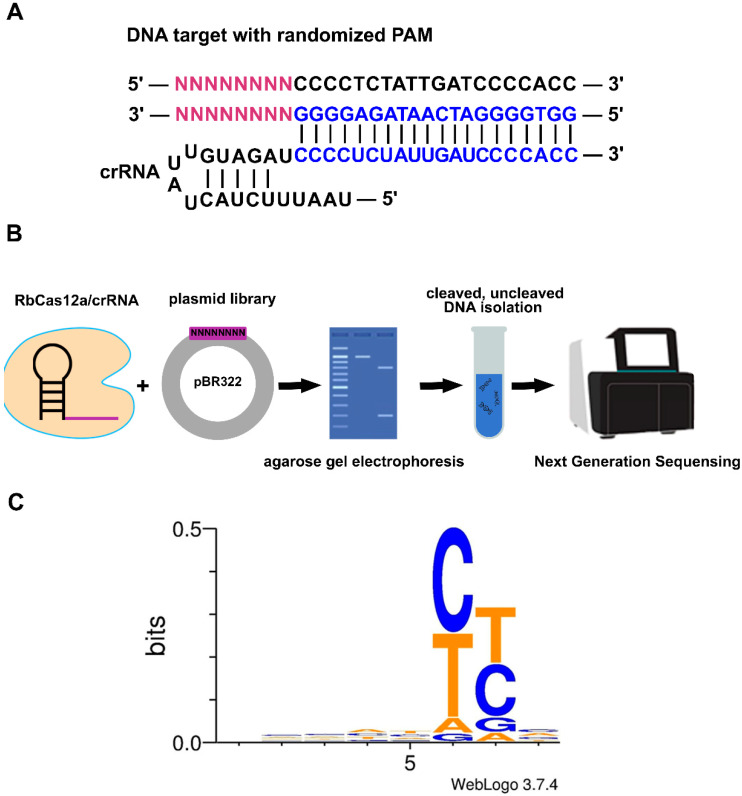
*Ruminococcus bromii* CRISPR–Cas Type V system PAM sequence logo determined by linear DNA PAM library sequencing. (**A**) Schematic of the RbCas12a crRNA–DNA-targeting complex. The target DNA contains eight random nucleotides at the 5′-end PAM region. (**B**) Schematic illustration of the experimental assay used to discover the RbCas12a PAM position and identity. (**C**) Sequence logo for the determined RbCas12a PAM.

Although 5′-TTTG PAM was found to be suitable for efficient DNA template cleavage, next, we investigated the PAM preferences of RbCas12a. We tested its ability to cleave linear DNA PAM libraries containing a target site flanked by eight randomized nucleotides at the 5′-end (Figure 3A). RbCas12a in a complex with crRNA was incubated with the PAM library at 37 °C for 1 h, and cleaved and uncleaved molecules were purified after agarose gel electrophoresis, and sequenced using the Illumina platform (Figure 3B). The bioinformatic analysis allowed us to determine the RbCas12a PAM logo. The data presented in Figure 3C shows that RbCas12a is capable of cleaving targets flanked by a 5′-YYN PAM.

**Figure 4:**
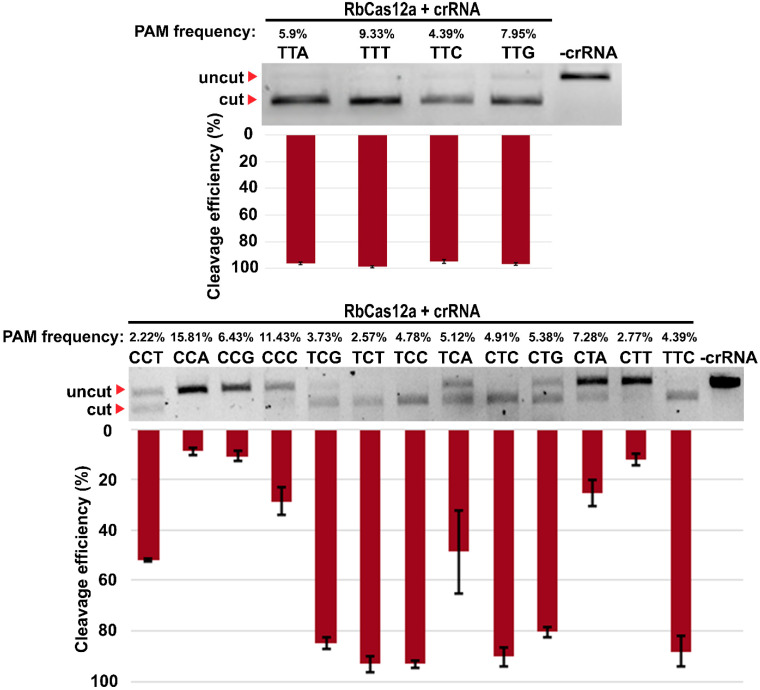
Investigation of RbCas12a PAM sequence by in vitro cleavage assay. The reactions contained 1.4 nM target DNA bearing the same protospacer but varying in the 5′-PAM sequence (as indicated above the panel), 346 nM recombinant RbCas12a, and 550 nM crRNA (a 0.1:35:55 nM ratio). Reactions were incubated for 30 min at 37 °C, and products were resolved by agarose gel electrophoresis. PAM frequency refers to the frequency of exact PAM in the cleaved PAM library. Mean cleavage efficiencies and standard deviations from three independent experiments are shown below the gel.

To further investigate RbCas12a PAM preferences, we created several corresponding PAM sites to cleave target DNA templates. We varied the four nucleotides, one at a time, at each of the three positions of the 5′-YYN PAM sequences. As shown in Figure 4 (upper panel), there was no preference for nucleotides in the N position of PAM. These experiments reveal that PAM position-1 was unrestricted for RbCas12a, while thymidine was disfavored by, for example, AsCas12a and LbCas12a nucleases. To investigate the effects of the first two nucleotides on cleavage efficiency, we tested the activity of the RbCas12a/crRNA complex with all possible 5′-YYN combinations but for 5′-TTD (where D represents T, A, or G). The results show that the efficiency of RbCas12a-mediated DNA cleavage of 5′-TCB and 5′-CTS was ~80% (where B represents C, T, or G; and S represents G or C), which is similar to 5′-TT(N) PAM variants (Figure 4, bottom panel). Much less efficient cleavages were detectable for the 5′-CCT and 5′- TCA PAMs (which were ~27%), whereas the 5′-CCA-, 5′-CCG-, and 5′-CTT-PAM-flanked protospacer was cleaved with just ~10%. Taken together, these results clearly demonstrate that RbCas12a requires a PAM defined as 5′-YYN for cleavage, at least in a pure in vitro system. It should be noted that 5′-CCV and 5′-CTW (where V represents C, A, or G; and W represents A or T) were not able to cleave DNA templates with efficiencies of more than 25% under the conditions of the experiment.

### 2.3. In Vitro Cleavage of DNA by RbCas12a

**Figure 5:**
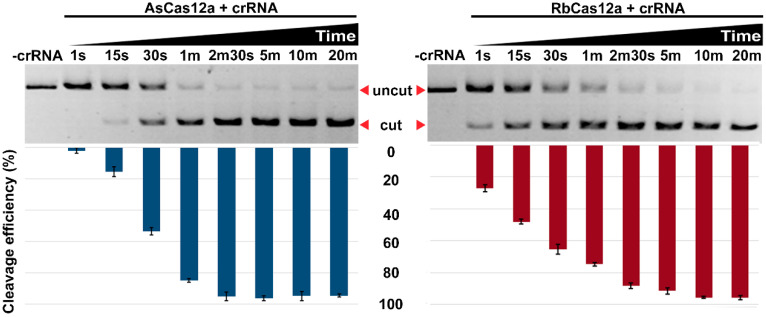
Effect of time on RbCas12a/crRNA and AsCas12a/crRNA cleavage activity. Target DNA cleavage by AsCas12a or RbCas12a programed with 500 nM crRNA at 1:3:30 target:Cas12a:RNA ratio. Mean cleavage efficiencies and standard deviations calculated from three independent experiments are shown.

Next, we investigated the kinetics of target DNA cleavage by RbCas12a or AsCas12a programed by appropriate crRNAs. As shown in Figure 5, in reactions containing the RbCas12a/crRNA complex, the target DNA was cleaved by ~27% after incubation for just 1 s. In the reaction containing the AsCas12a/crRNA complex, the target DNA was cleaved by ~15% only after 15 s of incubation. The cleavage reactions were ~50% complete after 15-s incubation with target DNA:RbCas12a:crRNA, and after 30-s incubation with target DNA:AsCas12a:crRNA. After 20-min incubation, both RbCas12a and AsCas12a cleaved ~95% of the target DNA under the experimental conditions. All subsequent experiments were performed using a 20-min incubation time interval. In the experiment, we used a DNA template with TTC PAM sequence.

**Figure 6:**
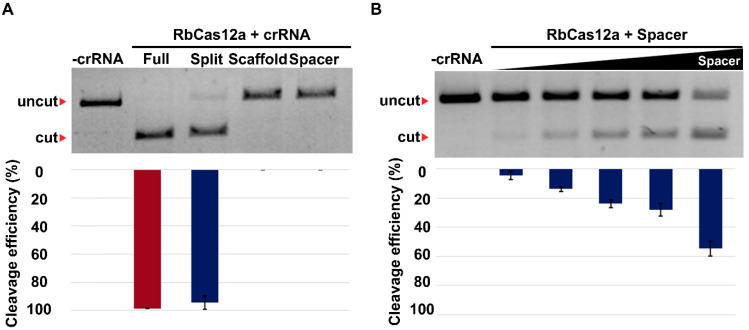
Effect of split crRNA and spacer-only RNA on RbCas12a cleavage activity. (**A**) Target DNA cleavage by RbCas12a programed with 0.3 μM full-sized split or separate scaffold and spacer crRNA moieties at a 1:3:30 target:RbCas12a:RNA ratio. “Split” reactions contained both the scaffold and spacer RNAs. Mean cleavage efficiencies and standard deviations calculated from three independent experiments are shown. (**B**) Cleavage by RbCas12a programed with increasing concentrations (0.5, 1, 2, 2.5, and 5 μM of spacer RNA, corresponding to 1:5:50, 1:5:100, 1:5:200, 1:5:250, and 1:5:500 DNA:RbCas12a:crRNA ratios). Mean cleavage efficiencies and standard deviations calculated from three independent experiments are shown.

Recently, we demonstrated that the cleavage activity of at least three V-A nucleases (AsCas12, LbCas12a, and FnCas12a) persists after a substantial reduction in the crRNA scaffold length, up to its complete removal, at high concentrations of crRNA. Furthermore, we showed that the trans-addition of a 20-base scaffold RNA rescued the cleavage activity of these enzymes. Thus, we proposed the concept of split crRNA, which, in a complex with V-A nucleases, was comparable to cleavage activity observed with full-sized crRNA [17]. Here, we expand our observations and investigate the ability of RbCas12 to be programed with split crRNAs. As shown in Figure 6A, in reactions containing both full-sized and split crRNAs, the target DNA was cleaved by ~90% under the conditions of the experiment. We previously showed that increasing the spacer-only crRNA concentration could increase the cleavage efficacy of AsCas12a [17]. The data presented in Figure 6B demonstrate that increasing the concentration of spacer RNA from 0.5 to 5 μM causes dose-dependent DNA substrate cleavage by RbCas12a. It is possible to cleave the DNA template reasonably efficiently (~50% after a 20-min incubation) at a protein:RNA ratio of more than 1:500. Efficient cleavage of cognate targets was also observed when split crRNAs with unrelated spacer sequences were tested (Figure S2).

### 2.4. Activity of RbCas12a at Different Temperatures

**Figure 7:**
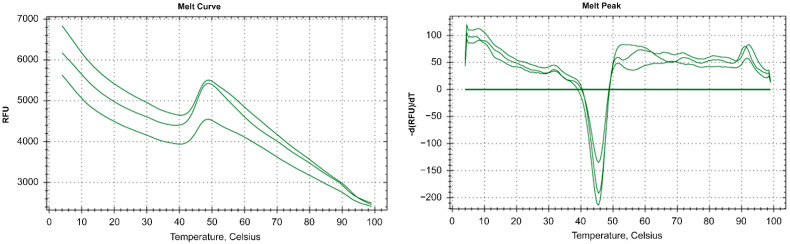
Thermofluor assay of RbCas12a. The left panel shows the melt curves, and the right panel shows the first derivative of the melt curves. The assay was performed with three independent replicas. The melt point of RbCas12a is 45.5 °C.

**Figure 8:**
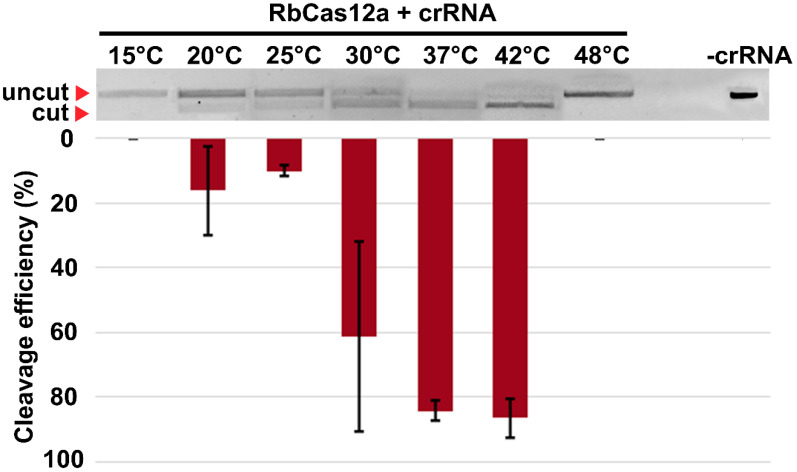
Effect of temperature on RbCas12a cleavage activity. Target DNA cleavage by RbCas12a programed with 500 nM crRNA at 1:3:30 target:Cas12a:RNA ratio. Mean cleavage efficiencies and standard deviations calculated from three independent experiments are shown.

In order to clarify the temperature range of RbCas12a activity, the stability of the enzyme in the NEBuffer 2 was measured by thermofluor assay. This fluorescent-dye-based method allowed us to examine the melting point of a protein under particular conditions. As shown in Figure 7, the Tm of RbCas12a was calculated as 45.5 °C. Next, in vitro cleavage reactions at different temperatures were performed, showing the inability of the ribonucleoprotein (RNP) complex to assemble and cleave dsDNA at temperatures higher than 45.5 °C, because of the process of unfolding RbCas12a and crRNA (Figure 8). In the experiment, we used a DNA template with a TTC PAM sequence. Surprisingly, nuclease efficiency was not affected at 42 °C compared to 37 °C. This might be explained by the environmental conditions of *Ruminococcus bromii*, as human gut temperature is slightly higher compared with other parts of the body. The RbCas12a RNP complex was able to cleave template DNA successfully even at 30 °C, although with reduced efficiency in some replicates, showing 61.3% cleavage efficiency (s.d. 29.2). RbCas12a was still able to cleave target DNA at lower temperatures, down to 20 °C, although with significantly less efficiency. It is important to note that, according to our data, RbCas12a RNP complex formation is unstable at 20 °C, showing 5.4% to 35.5% cleavage efficiency, which means that the particular temperature point is close to the lowest temperature limit of the protein activity in vitro. As expected, we did not observe any DNA cleavage at 15 °C, confirming that RbCas12a cannot efficiently bind to crRNA. Nevertheless, successful RNP complex formation and subsequent efficient cutting events depend not only upon temperature conditions, but also the particular spacer and PAM sequences.

### 2.5. RbCas12a Nuclease Is Active in Human Cells

We tested RbCas12a activity in human cells. A codon-optimized RbCas12a gene was cloned into plasmid vectors under the regulation of the CMV promoter. Three nuclear localization sequences (NLS) were added to the N-end of the protein, and one to the C-end, to enhance its import to the nucleus after translation. Appropriate crRNA coding sequences were cloned into independent plasmid vectors under the control of the U6 promoter. RbCas12a was targeted to the human *DNMT1*, *EMX1*, and *VEGFA* genes. We also checked the presence of nucleases via RT-PCR (Figure S3). Across the three genes assessed, there were no statistical differences (*p* > 0.05) with control samples (without crRNA/RbCas12a transfection) under the conditions of the experiment.

**Figure 9:**
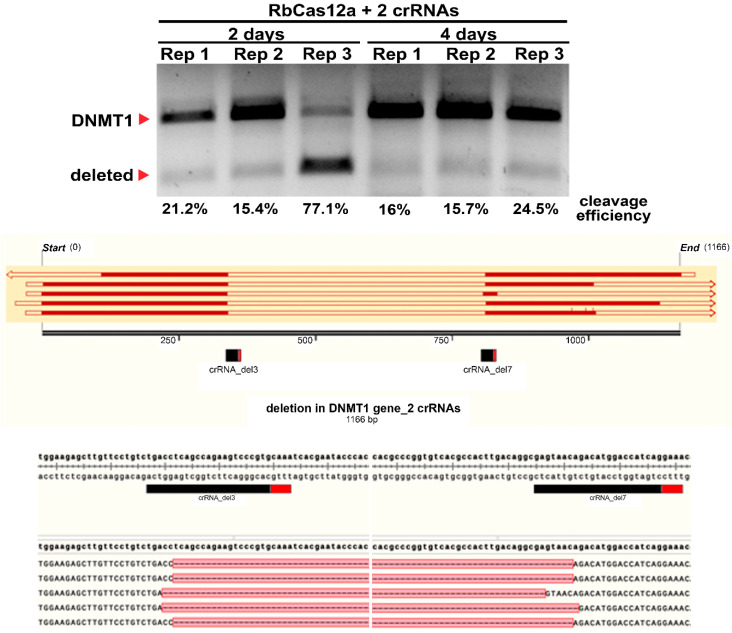
Targeting the human *DNMT1* gene with two crRNAs led to deletions. Top: Agarose gel visualization of PCR products after cell culture transfection with plasmids encoding RbCas12a and two crRNAs. Three independent experiments are shown as Rep1–3 on the second and fourth days of the experiment. Middle: A schematic representation of the PCR product with two guide RNAs highlighted. Bottom: Detailed maps with nucleotide sequences, created with SnapGene software.

We theorize that, in the absence of detectable target cleavages in cells transfected with separate crRNA, using two different crRNAs simultaneously could produce results. To test this assumption, two different crRNAs simultaneously targeting the human *DNMT1* gene were used. As shown in Figure 9, we detected a band corresponding to the deleted DNA fragment after PCR. The efficiencies of the repeats were ~15–25%, except in one case, where ~77% was obtained. We performed Sanger sequencing on several colonies that hosted cloned PCR fragments, and detected breaks approximately −16–19 nts upstream of the PAM for both crRNAs under the experimental conditions (Figure 9, bottom panel). Encouraged by these high efficiencies, we decided to achieve a more detailed picture of break site flanking deletions by performing NGS on deleted PCR products for both test days.

**Figure 10:**
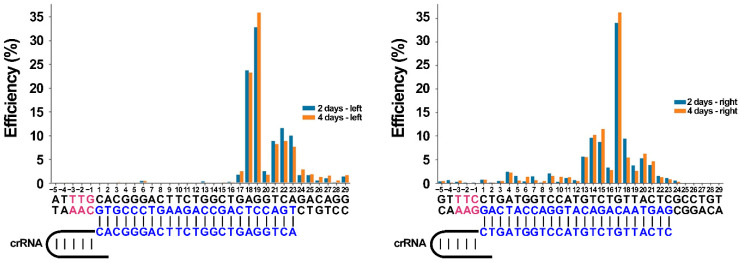
Frequencies of break site flanking deletion. On the left side of the figure, a crRNA is indicated, which, complexed with RbCas12a, forms break sites flanking the left arm of the deletion. On the right side of the figure, the right crRNA is indicated. PAM sequences on both target and nontarget strands are highlighted in red font. The protospacer position on the target DNA strand and spacer moiety of crRNA is highlighted in blue font. Relative coverage depth in the edited region was calculated as depth coverage in certain positions divided by mean coverage depth outside of the edited region. DNA break efficiency after a certain position was assessed as the difference between relative depth in two neighboring positions.

We must clarify what we mean by “break sites”. As each NGS read consists of both deleted protospacer DNA regions linked to each other, we call the link sites “break sites”. That is, the place of transition from one protospacer to another. As can be seen from Figure 10, the most common break sites were between 19 and 20 nts within the protospacer region of the left arm of deletions, and between 17 and 18 nts within the protospacer region of the right arm of deletions. The results show that the efficiency of the aforementioned break sites was ~35% for both left and right crRNAs at the conditions of the experiment. It is interesting to note that the distribution of the break sites inside the sequence protospacer for the right crRNA appears unimodal, while it appears bimodal for the left one. This might be a consequence of cleavage efficiency of the DNA template by the crRNA/RbCas12a complex. Another peak value for left crRNA break sites was ~23%, and localized between 18 and 19 bps. The cleavage reactions were ~10% complete for both left and right crRNAs, but at different sites: after 21, 22, and 23 bps inside the sequence protospacer for the left crRNA, and after 14 and 15 bps for the right one. There was no significant difference (chi-squared test *p*-value > 0.05) in the break efficiencies between the second and fourth days of the experiment. There was also no statistical relationship between the first and second break sites in the experiment. These results indicate that RbCas12a is active in human cells.

## 3. Discussion

The CRISPR–Cas12 subtype V-A system (Cas12a), also known as Cpf1, is a genome editing tool that is considered as an alternative to Cas9 nucleases. Although SpCas9 is still more commonly used than nucleases of the Cas12a family, the latter have gained momentum in medical practice and breeding biotechnologies, mainly due of their smaller size in terms of both nucleases and crRNA. Here, we present a novel RNA-guided DNA endonuclease from *Ruminococcus bromii*, which belongs to the Cas12a subtype. It was assigned to the Cas12a family according to four independent lines of evidence: (i) RbCas12a is clustered with other Cas12a enzymes in the phylogenetic tree; (ii) the scaffold of the crRNA is similar to those of previously published Cas12a orthologs; (iii) the PAM is similar to those of other Cas12a orthologs, and consists of ‘YYN’ nucleotides; (iv) the amino acid sequence of RbCas12a shares the same conservative catalytic sites with other Cas12a orthologs.

As previously mentioned, Cas12a orthologs have been characterized, among others, as having very similar structures among the crRNA scaffolds, which differ only in the loop region (Table S1). Among the 19 Cas12a orthologs known to us that can cleave chromatin in human cells, there are ones that possess three, four, or five nucleotides in the loop region of the crRNA. For instance, AsCas12a and ErCas12a crRNA loops consist of UCUU nucleotides. FnCas12a, ArCas12a, BfCas12a, and TsCas12a possess UGUU nucleotides, and MbCas12a possesses UUUU nucleotides. CeCas12a is the only known Cas12a ortholog capable of editing the human genome, whose four-nucleotide crRNA loop region does not contain two terminal uridines, but a CG-dinucleotide instead. The loop region of crRNA for BsCas12a, HkCas12a, PxCas12a, Lb2Cas12a, and RbCas12a, reported here, consists of UAUU nucleotides. The only crRNA, to our knowledge, consisting of three UUU nucleotides in the loop, forms an RNP complex with the EeCas12a nuclease. The longest loop regions that are composed of five nucleotides, UGUGU, UGUGU, and UAAGU, are inherent in LpCas12a, PrCas12a, and LbCas12a, respectively. A similar UGUUU loop region is typical for Mb2Cas12a and Mb3Cas12a crRNAs, of which both were found in different strains of *Moraxella bovoculi* sp. It is worth noting that the similarity of the loop region of crRNA is not correlated with the phylogenetic affinity (grouped on the nearest branches) of amino acid sequences of Cas12a nucleases (Figure 1B). RbCas12a programmed with crRNA matched to As/ErCas12a is still able to cleave target DNA template (data not shown). This means that point mutations in the loop do not affect the protein binding activity of crRNA, as this region does not participate in ribonucleoprotein interactions. Thus, the diversity of loop sequence variants can be explained by an accumulation of random mutations that are not subject to targeted evolutionary selection.

To comprehensively characterize the in vitro conditions of the target DNA cleavage by RbCas12a, we harnessed a set of divalent cation-contained salts supplemented into the reaction buffer. Recently, it was reported that the presence of manganese ions, AsCas12a, in contrast to LbCas12a, almost completely degrades dsDNA in the absence of crRNA [18]. Our data show that the RbCas12a RNP complex is able to utilize magnesium as well as manganese ions for efficient crRNA-guided dsDNA cleavage without nonspecific degradation of the matrix. Moreover, zinc and nickel can act as cofactors for RbCas12a, although with reduced efficiency. Interestingly, calcium did not support the RbCas12a binding and cleavage activity, in contrast to previously reported data on FnCas12a [19]. In summary, these results confirm the thesis stated in [18], that diverse Cas12a orthologs possess different preferences for metal ions.

We further characterized the RbCas12a RNP complex formation possibility and its targeting efficiency over a relatively wide range of temperatures, from 20 °C to 42 °C. In contrast to previously reported approaches [20], we incubated separate contents of reactions at appropriate temperatures in different tubes, thus allowing crRNA, protein, and target DNA structure formation corresponding to the particular temperature point. After that, crRNAs were mixed with RbCas12a at the same temperature in a preincubation step, which could help determine whether the RNP complex is to be, or not to be, assembled. In the end, a potentially contained RNP solution was supplemented with DNA exhibiting EDTA inactivation after incubation. Since every step in these experiments was performed at appropriate temperatures, the results might be considered as more closely reflecting native properties of nucleases. It should be noted that, in the experiments conducted at routine room temperatures, mixing of reaction contents followed by incubation at appropriate temperatures resulted in dsDNA cleavage in a wide range of conditions, from 4 °C to 100 °C, most likely due to the rapid rate of cleavage (data not shown). Nevertheless, one has to take into account that this data should not be considered as results reflecting the in vivo activity of the nucleases, but can be applied to a number of in vitro applications of CRISPR/Cas12a-based methods. Moreover, the cleavage rates presented here and in similar studies indicate the results of particular experiment conditions (cleavage buffer; RNA:protein:DNA ratio; spacer and PAM sequences, i.e., specificity of particular crRNA), and should not be translated to other investigations. Despite these limitations, the aforementioned experiments may help us to understand the nature of the enzyme and its activity in vitro more clearly. In addition, as biotechnological and agricultural demand for enzymes that are active at lower temperatures constantly grows, we suppose that relatively efficient cleavage at up to 20 °C can present an opportunity for further studies focusing on plant genome editing, as such experiments are still very sensitive to temperature conditions.

In numerous reports, it was shown that similar, but different in detail, 5′T-rich PAM sequences are required for distinct Cas12a nucleases. In spite of this fact, and the published efforts on broadening PAM accessibility [21,22,23], it still remains a challenge to target not just desirable, but any T-rich locus of a genome. Results of the experiments from PAM studies show that AsCas12a and LbCas12a have sequences of 5′-TTTV, while those for FnCas12a and MbCas12a are both 5′-TTN [10]. In addition to the canonical PAM sequences, Cas12a also exhibits relaxed PAM recognition for suboptimal C-containing PAMs by forming altered interactions with the targeted DNA duplex. The presented novel nuclease RbCas12a recognizes almost all 5′-YYN PAMs, which, to the best of our knowledge, is the least stringent PAM reported to date. The reader may find the logarithmic-scale visualization of this finding in Figure 4 and Figure S4.

Despite the advantages of RbCas12a among known orthologs, there are still several reasons why Cas12a cannot completely replace Cas9 in most genome editing techniques. First of all, engineered state-of-the-art Cas9 nucleases are almost PAM-independent, whereas Cas12a is yet to be modified to target all Cas9-accessible loci. Second, Cas12a proteins show reduced activity in relation to nucleosome-occluded genome regions, and require nucleosome unwrapping agents, such as dCas9, to access the targeted locus [24]. Finally, Cas9 nickases are used for precise genome modifications, bypassing DSBs and HDR through prime editing. No Cas12a nickase with proven activity in eukaryotic cells has been engineered to date. It was recently published that a Cas12a_M3 variant nicks the target strand of DNA in vitro without DSB generation, although in a sluggish manner [25]. Further studies will show the effectiveness of Cas12a_M3 or its derivatives in vivo, and will possibly pave the way for the engineering of nickase-based Cas12a prime and base editors. Overcoming the highlighted challenges will put Cas12a on a par with Cas9 in genome editing applications.

According to previously published data, Cas12a/crRNA complexes can differ in cleavage efficiency in both in vitro and in vivo experiments. Chromatin accessibility seems to be one of the reasons for these differences, although this requires additional investigation. In this work, we show that in spite of the absence of genome editing traces when using one crRNA, it is still possible to utilize the CRISPR/Cas12a nuclease for targeted genome deletion incorporation. Using two crRNAs simultaneously increases the cleavage efficiency by incorporating at least a 471 bp deletion, in our case. A number of publications examining whether one Cas12a ortholog or the other can be applied for mammalian genome editing report the absence of discovered mutations after cell culture transfections using the T7E1 assay. Here we provide evidence that these observations in some cases can be false-negative. To the best of our knowledge, there is only one protocol for Cas12a utilization, reported by the Jinek group [26], describing ~500 bp deletion incorporation in mouse ES cells, transfected by RNP charged with two different crRNAs. In the present study, we demonstrate the ability to use another Cas12a ortholog. With great efficiency in creating the deletion, we co-transfected the HEK293T human cell line with three plasmid vectors, one of which encoded the nuclease and two encoded other crRNAs.

Our data on the crRNA/RbCas12a help to extend the opportunities of genome editing. The principal finding of this paper is the demonstration that the RbCas12a effector can be used for human genome deletion formation with high efficiency. It is interesting to note that the absence of detectable cleavage when using only one crRNA does not mean the absence of the functional activity of the effector. Carefully and effectively created deletions could be used for cell model creation for human diseases caused by fragment deletions. The main limitation of the present article is that proof of suitable PAM patterns and expanded range of temperature in a pure was demonstrated only in an in vitro system. The effectiveness of deletion creation should also be checked using different PAMs and temperatures. Future research on the current topic needs to uncover the off-target effects of the new RbCas12a ortholog, and compare the results with other widely used Cas12a effectors.

## 4. Materials and Methods

### 4.1. Database Mining for Nuclease Identification

Mining of the NCBI nr/nt database resulted in the identification of RbCas, possessing the typical Cas12a domain structure, however, showing only 46.26% identity to the closest experimentally characterized LbCas12a from *Lachnospiraceae bacterium* ND2006.

### 4.2. Phylogenetic Tree Reconstruction

In total, 18 Cas12a proteins functioning in mammalian cells and their close orthologs (HkCas12a, *Helcococcus kunzii* ATCC 51366; AsCas12a, *Acidaminococcus* sp. BV36L; LpCas12a, *Lachnospira pectinoschiza* strain 2789STDY5834886; CeCas12a, *Coprococcus eutactus sp*.; EeCas12a, *Lachnospira eligens* ATCC 27750; ArCas12a, *Agathobacter rectalis* strain 2789STDY5834884; ErCas12a, *Eubacterium rectale* sp.; FnCas12a, *Francisella novicida* U112; TsCas12a, *Thiomicrospira* sp. XS5; MbCas12a, *Moraxella bovoculi* 237; Mb2Cas12a, *Moraxella bovoculi* AAX08_00205; Mb3Cas12a, *Moraxella bovoculi* sp.; BfCas12a, *Butyrivibrio fibrisolvens* MD2001; PrCas12a, *Pseudobutyrivibrio ruminis* CF1b; PxCas12a, *Pseudobutyrivibrio xylanivorans* DSM 10317; BsCas12a, *Butyrivibrio* sp. NC3005; Lb2Cas12a, *Lachnospiraceae bacterium* MA2020; LbCas12a, *Lachnospiraceae bacterium* ND2006; RbCas12a, *Ruminococcus bromii* sp.) [13] were selected for multiple sequence alignment with RbCpf1. The sequences were aligned using MAFFT v.7.475 [27] with L-INS-i algorithm and BLOSUM62 evolutionary model. The maximum-likelihood phylogenetic tree was reconstructed using IQ-TREE2 v.2.1.2 [28]. Evolutionary model LG + F + I + G4 was automatically selected using ModelFinder and Bayesian Information Criterion; 1000 bootstraps were performed.

### 4.3. Plasmid Cloning

To confirm the presence of the *cas12a* gene in the samples, PCR was performed with the primers Fwd_test and Rev_test (sequences are listed in Table S2). Fifty microliters of the reaction mixture were mixed using Q5^®^ High-Fidelity 2X Master Mix (New England Biolabs, Ipswich, MA, USA), according to the manufacturer’s instructions. Three of the seven samples that contained target fragments (~590 bp) were further utilized in PCR reactions to amplify the whole gene with the 5′-phosphorylated primers RbCpf_Fwd_Nde and RbCpf_Rev_Xho. Target fragments were excised from agarose gel, and DNA was extracted using the QIAGEN Gel Extraction Kit (Qiagen, Hilden, Germany) according to the manufacturer’s instructions. Next, *cas12a*-containing fragments were cloned into the linearized pUC118 vector (HincII endonuclease, QuickCIP, and T4 DNA ligase, New England Biolabs, Ipswich, MA, USA). Plasmids were transformed into *E. coli* TOP10 strain and screened by PCR the next day. Any positive colonies were inoculated into Luria–Bertani media for overnight growth at 37 °C. Plasmids were extracted with a Monarch^®^ Plasmid Miniprep Kit (New England Biolabs, Ipswich, MA, USA) and sequenced on Illumina MiSeq (Illumina, San Diego, CA, USA). Next, *cas12a* genes from three samples were cloned into the pET30a+ vector using NdeI and XhoI sites and further transformed, as previously described. To confirm successful assembly, the extracted plasmid DNA was restricted and analyzed on agarose gel. Human and plant codon optimization was performed by DAPCEL, Inc. (Cleveland, OH, USA) by the selection of synonymous codons to ensure the correct co-translational folding of the proteins in a target host. The plasmid vector containing hRbCas12a in the pcDNA3.1(+) backbone was created by GenScript LCC (Piscataway, NJ, USA). To confirm the successful import of the nuclease into the nucleus, three additional nuclear localization signals (NLSs) were introduced into the N-terminus of human-optimized proteins by sequential restriction–ligation steps using HindIII restriction endonuclease (New England Biolabs, Ipswich, MA, USA). Briefly, two SV40-NLS-containing complementary oligonucleotides with scattered phosphorylated ends were annealed and subsequently ligated into HindIII-linearized and dephosphorylated hRbCas12a-pcDNA3.1(+). All crRNA-coding plasmid vectors for human cell culture were constructed as follows: first, the RFP gene under the control of the CMV promoter was cloned into pSQT1313 obtained from Addgene (#53370). Next, the RbCas12a crRNA scaffold with two BsmBI sites was cloned using site-directed mutagenesis. All spacers were then cloned at the BsmBI sites by annealing two complementary oligonucleotides with staggered ends.

### 4.4. Recombinant Protein Purification

For recombinant products, the *E. coli* Rosetta strain was transformed with the plasmid DNA, and clones were grown in Luria–Bertani (LB) medium at 37 °C overnight. The next morning, bacterial suspensions were inoculated into fresh LB medium and cultivated for 3 h at 37 °C, followed by cooling to room temperature. Then, IPTG was added, and the cells were incubated at 18 °C overnight. The cell lysate was loaded onto a Ni-NTA Superflow 5-mL column (Qiagen, Hilden, Germany) and equilibrated with a buffer containing 5 mM imidazole. The recombinant protein was eluted with a buffer containing 300 mM imidazole and the buffer was exchanged with 50 mM phosphate buffer (pH 7.4). Then, the protein was applied to MonoS 10/100 (GE Life Science, Carlsbad, CA, USA) and eluted with a linear gradient of NaCl in the same buffer. Fractions containing RbCas12a were collected, concentrated to ∼12 mg/mL, and snap-frozen with liquid nitrogen in small aliquots that were stored at −80 °C until use. The protein concentration was measured using the Qubit Protein Assay Kit (Thermo Fisher, Carlsbad, CA, USA), according to the manufacturer’s instructions. Protein quality was assessed by SDS-PAGE and visualized by Coomassie blue staining.

### 4.5. PAM Identification

Two M13-flanked, reverse-complemented 100-bp oligos with 8-bp degenerate fragments (Sintol, Moscow, Russia) adjacent to the protospacer were utilized as templates in the independent PCR reactions (Q5 DNA Polymerase; New England Biolabs, Ipswich, MA, USA). Amplicons were analyzed by agarose gel electrophoresis followed by gel extraction using a Qiagen Gel Extraction Kit (Qiagen, Hilden, Germany). The resulting fragments were restricted by EaeI (New England Biolabs, Ipswich, MA, USA) and cloned into the pBR322 vector linearized by EagI and PvuII (New England Biolabs, Ipswich, MA, USA), followed by transformation into the *E. coli* TOP10 strain. Since EaeI and EagI form compatible sticky ends, the fragment’s insertion orientation was determined. Sixteen hours after transformation, more than 180,000 colonies were washed off the plates, and the plasmid library was extracted using the GenElute™ HP Plasmid Maxiprep Kit (Sigma, Merck KGaA, Darmstadt, Germany). Next, the obtained pDNA was cut using RbCas12a and gel-analyzed. Reactions were incubated for 30 min at 37 °C, and products were resolved by agarose gel electrophoresis. Cut and uncut fragments were eluted from the gel and used as an input for fragment library preparation with the Ultra II Library Prep kit (New England Biolabs, Ipswitch, MA, USA). The resulting libraries were checked using the Bioanalyzer 2100 instrument (Agilent, Santa Clara, CA, USA) and sequenced with the MiSeq™ instrument (Illumina, San Diego, CA, USA) using 250-bp paired-end reads. A total of 517 thousand raw read pairs were obtained and subjected to stringent quality trimming with CLC Genomics Workbench v.20.0 (Qiagen, Hilden, Germany). The trimming parameters were as follows: the quality score limit of error probability was 0.01, and of allowed ambiguities was 0. Trimmed overlapping reads were merged with SeqPrep tool (https://github.com/jstjohn/SeqPrep (accessed on 15 July 2021)) using default parameters, resulting in approximately 480 thousand merged reads. For the alignment, only the reads containing an exact 20-nt match to PAM-flanking sequences were used (flanking motif search was performed with the seqkit grep command of the SeqKit package). The final alignment contained 180 thousand reads. The flanking sequences of the PAMs were trimmed from the alignment using CLC Genomics Workbench (Qiagen, Hilden, Germany), and the trimmed alignment was used as an input for the WebLogo server.

### 4.6. Checking Different PAM Sequences to Run the Protospacer Cleavages

To create DNA templates with different PAMs but the same protospacer, we used a site-directed mutagenesis (SDM) approach. The initially constructed PAM TTG was mutated in three ways, resulting in all four possible TTN variants for the same protospacer. SDM was performed using the Q5^®^ Site-Directed Mutagenesis Kit (New England Biolabs, Ipswich, MA, USA), according to the manufacturer’s instructions, with the primers listed in Table S2. Successful amplification was verified by performing electrophoresis on a 1% 1× TAE agarose gel. Plasmid DNA was extracted using the Monarch^®^ Plasmid Miniprep Kit (New England Biolabs, Ipswich, MA, USA). To confirm the authenticity of the PAMs identified by NGS sequencing and WebLogo representation (see Results), pUC119 plasmid vector was cleaved using 13 different crRNAs targeting protospacers with YYN PAMs: 12 crRNAs targeting CTN and TCN PAMs, and TTC-targeting crRNA as the control. The oligonucleotides used in this study are listed in Table S2. All crRNAs were obtained by in vitro transcription using the HiScribe™ T7 High Yield RNA Synthesis Kit (New England Biolabs, Ipswich, MA, USA). Briefly, 53 pmol complementary oligonucleotides were equimolar mixed, heated at 95 °C for 5 min, incubated at room temperature for 30 min, and subsequently used as a matrix. Overnight RNA synthesis was performed with 10 mM final concentration of each NTP in 30 µL reactions, followed by DNAse I treatment. Obtained crRNAs were then extracted using Trizol (Thermo Scientific, Carlsbad, CA, USA) according to the manufacturer’s instructions. RNA concentration was measured on Qubit 3.0 using a Qubit RNA HS Assay Kit (Thermo Scientific, Carlsbad, CA, USA).

### 4.7. In Vitro DNA Cleavage Assays

In vitro cleavage assays with the purified RbCas12a nuclease were carried out in a volume of 30 µL in 1× NEBuffer 2 (New England Biolabs, Ipswich, MA, USA). Next, 10 nM PCR-obtained DNA template, 500 nM of the nuclease, and 5000 nM crRNA were incubated for 30 min at 37 °C, and the reaction was stopped by the addition of Proteinase K (New England Biolabs, Ipswich, MA, USA) at a final concentration of 1 µg/µL, followed by incubation at room temperature for 15 min. Reaction analysis was performed by electrophoresis on a 1% agarose gel in TBE buffer. EtBr prestaining gels were visualized using a Gel Doc EZ Imager (Bio-Rad) and quantified with Image Lab™ Software v.6.0.1 (Bio-Rad, Hercules, CA, USA). DNA substrates for in vitro cleavage assays represent fragments of DNA amplified by PCR in Q5^®^ High-Fidelity 2X Master Mix (New England Biolabs, Ipswich, MA, USA). Genomic DNA was isolated from the T-REx cell line (T-REx™-293 Cell Line, #R71007; Invitrogen, Carlsbad, CA, USA) using the GeneJET Genomic DNA Purification Kit (Thermo Scientific, Carlsbad, CA, USA). DNA concentration was measured on a Qubit 3.0 fluorometer using a Qubit DNA BR Assay Kit (Thermo Scientific, Carlsbad, CA, USA). Oligonucleotide primers and RNA moieties provided by Lumiprobe RUS Ltd. (Moscow, Russia) are listed in Supplementary Table S2. For the study of divalent cation impact on RbCas12a activity, 900 pM of linear dsDNA bearing 5′TTC PAM, 550 nM crRNA, and 350 nM protein were mixed in the cleavage buffer (containing 10 mM Tris-HCl (pH 7.9) and 50 mM NaCl), and supplemented with 10 mM of either CaCl2, MgSO4, MnCl2, ZnSO4, Ni(NO3)2, CuSO4, or CoCl2 (Sigma-Aldrich, Merck KGaA, Darmstadt, Germany), followed by incubation for 30 min at 37 °C. Reactions were stopped by the addition of 100 mM EDTA, and run in 1xTAE agarose gel electrophoresis with ethidium bromide prestaining.

### 4.8. Thermostability Assay and Activity at Different Temperatures

To investigate RbCas12a thermostability, a fluorescent-based assay was performed using ProteOrange Protein Gel Stain 5000× (Lumiprobe RUS Ltd., Moscow, Russia) under the following conditions: we used 9 µg purified RbCas12a, 12.5 µL NEBuffer 2 (New England Biolabs, Ipswich, MA, USA), and 2.5 µL ProteOrange Protein Gel Stain (diluted to 50×) in 25-µL reactions. A thermal curve was constructed for both the negative control (without protein) and reactions containing RbCas12a (each in triplicate) from 4 °C to 95 °C, with increments of 0.5 °C and signal registration every 10 s. To determine the temperature dependence of RbCas12a\crRNA complex formation, and consequently, RbCas12a activity, in vitro cleavage reactions were carried out to completion at the point temperatures. First, 225 nM RbCas12a, 375 nM crRNA, and 500 pM DNA bearing 5′-TTC PAM in 1x NEBuffer 2 were first incubated in separate tubes at corresponding temperatures for 5 min. Next, ribonucleoprotein (RNP) complexes were obtained by mixing RNA and RbCas12a, followed by incubation at the same temperature for 15 min. RNP complexes were further supplemented with DNA, followed by an additional 20-min incubation step. Last, 83 mM EDTA was added to the reactions for complete inactivation of the enzyme. Cleavage efficiency was analyzed by agarose gel electrophoresis in TAE.

### 4.9. Human Cell Culture and Transfection

T-REx™-293 cells were cultivated in Dulbecco’s modified Eagle medium (Thermo Fisher, Carlsbad, CA, USA) with 10% fetal bovine serum (Thermo Fisher Scientific, Carlsbad, CA, USA) and 1% penicillin–streptomycin (Thermo Fisher, Carlsbad, CA, USA) at 37 °C with 5% CO2 until 85% confluence. The transfection, which was 1 day following cell plating, was performed in 6-well plates using Lipofectamine 3000 Reagent (Thermo Fisher, Carlsbad, CA, USA), according to the manufacturer’s protocol. A total of 2000 ng hRbCas12a plasmid and 2000 ng crRNA plasmid were used to target the *DNMT1*, *VEGFA*, or *EMX1* genes. *DNMT1* was targeted using either one crRNA plasmid or two plasmids (each crRNA encoded in separate plasmids), with the latter aimed at obtaining a respective deletion. At 48 h after transfection, cells were washed three times with PBS and lysed with NP40 Cell Lysis Buffer (Invitrogen, Carlsbad, CA, USA) containing 0.2 mg/mL Proteinase K. Cells transfected with both plasmids were processed either 2 days or 4 days after transfection.

### 4.10. RbCas12a mRNA Detection by RT-PCR

Two days after transfection, the cells were harvested, washed with PBS, and total RNA was extracted with TRIzol (Thermo Fisher, Carlsbad, CA, USA). cDNA was obtained using the ProtoScript^®^ II First Strand cDNA Synthesis Kit (New England Biolabs, Ipswich, MA, USA). Real-time PCR amplification of cDNA was carried out in a 20-μL reaction mixture containing 10 μL EvaGreen Supermix (Bio-Rad, Hercules, CA, USA), 500 nM of each primer, 100 ng cDNA, and RNase-free water. The thermal cycling conditions for cDNA used in the CFX96 thermocycler (Bio-Rad, Hercules, CA, USA) were as follows: 95 °C for 3 min, 39 cycles at 95 °C for 10 s, 56 °C for 5 s, and 68 °C for 10 s.

### 4.11. Indel and Mismatch Frequency Analysis

The reads were mapped to the reference sequences using minimap2 v.2.19-r1057 [29] in short-read mapping mode. Coverage depth at certain positions was measured using samtools v.1.12 [30]. Relative coverage depth in the edited region was calculated as depth coverage in certain positions divided by mean coverage depth outside of the edited region. DNA break efficiency after a certain position was assessed as the difference between relative depth in two neighboring positions. The chi-squared test was used to determine the significance of the discrepancy between 48- and 96-h experiments. Mismatch and deletion frequencies in experiments with a single crRNA was assessed using bam-readcount v.1.0 (https://github.com/genome/bam-readcount (accessed on 13 September 2021)) with default settings.

### 4.12. Statistics

All statistical analyses were performed in R language (v.4.1.2). The exact replication numbers are indicated in the figure legends. The findings in all the figures of the gel images were successfully reproduced under similar experimental conditions at least three times independently. Differences were considered significant at the level of *p* < 0.05.

## Data Availability

All data presented in the manuscript is available upon request.

## Supplementary Materials

The following supporting information can be downloaded at: https://www.mdpi.com/article/10.3390/ijms23169289/s1.
