# Supplementary material for: Targeted Modification of Mammalian DNA by a Novel Type V Cas12a Endonuclease from Ruminococcus bromii
Source: Int J Mol Sci. 2022 Aug 18;23(16):9289. doi: 10.3390/ijms23169289 (PMC9409102; doi:10.3390/ijms23169289)
Supplement: Supplementary file 1 [file ijms-23-09289-s001.zip › ijms-1847072-supplementary.pdf]

SUPPLEMENTARY MATERIALS

Supplementary Table S1. Cas12a orthologs and their ability to edit the genomes of human cells

Cas12a orthologs	Short title	Scaffold nucleotide sequence, 5'-3'	Paper where was showed human cell gene editing, doi
Lachnospira eligens ATCC 27750	EeCas12a	UAAUUUCUACU UUGUAGAU	10.1038/ s41598-019-50423-6
Moraxella bovoculi 237	MbCas12a	UAAUUUCUACU **UUU**GUAGAU	10.1093/nar/gky815
Eubacterium rectale	ErCas12a	UAAUUUCUACU **CUU**GUAGAU	10.1089/crispr.2019.0026
Acidaminococcus sp	AsCas12a	UAAUUUCUACU **CUU**GUAGAU	10.1093/nar/gky815
Ruminococcus bromii sp.	**RbCas12a**	UAAUUUCUACU **AUU**GUAGAU	**current study**
Butyrivibrio sp. NC3005	BsCas12a	UAAUUUCUACU **AUU**GUAGAU	10.1186/ s13059-019-1620-8
Lachnospiraceae bacterium MA2020	Lb2Cas12a	UAAUUUCUACU **AUU**GUAGAU	10.1096/fj.202001013RR
Helcococcus kunzii ATCC 51366	HkCas12a	UAAUUUCUACU **AUU**GUAGAU	10.1186/ s13059-019-1620-8
Pseudobutyrivibrio xylanivorans strain DSM 10317	PxCas12a	UAAUUUCUACU **AUU**GUAGAU	10.1186/ s13059-019-1620-8
Francisella novicida	FnCas12a	UAAUUUCUACU **GUU**GUAGAU	10.1093/nar/gky815
Thiomicrospira sp. XS5	TsCas12a	UAAUUUCUACU **GUU**GUAGAU	10.2302/kjm.2019-0009-OA
Agathobacter rectalis strain 2789STDY5834884	ArCas12a	UAAUUUCUACU **GUU**GUAGAU	10.1186/ s13059-019-1620-8
Butyrivibrio fibrisolvens MD2001	BfCas12a	UAAUUUCUACU **GUU**GUAGAU	10.1186/ s13059-020-01989-2
Lachnospiraceae bacterium ND2006	LbCas12a	UAAUUUCUACU**AAG**UGUAGAU	10.1093/nar/gky815
Moraxella bovoculi AAX08_00205	Mb2Cas12a	UAAUUUCUACU**GUU**UGUAGAU	10.2302/kjm.2019-0009-OA
Moraxella bovoculi sp.	Mb3Cas12a	UAAUUUCUACU**GUU**UGUAGAU	10.1126/science.aau5174
Coprococcus eutactus sp.	CeCas12a	UAAUUUCUACU **UCG**GUAGAU	10.1186/ s13059-020-01989-2
Lachnospira pectinoschiza strain 2789STDY5834886	LpCas12a	UAAUUUCUACU**GUG**UGUAGAU	10.1186/ s13059-019-1620-8
Pseudobutyrivibrio ruminis CF1b	PrCas12a	UAAUUUCUACU**GUG**UGUAGAU	10.1186/ s13059-019-1620-8

Supplementary Table S 2. Sequence of oligonucleotides used in the study (T7 promoter complementary region of oligonucleotides is highlighted green, protospacer complementary region is highlighted red, scaffold is black)

**DNA oligonucleotides 5'->3'**	
Fwd_test	GGATGCAACTGAATCCTGTAG
Rev_test	TACCGCATAAGCCGGAATAAG
Fwd_RbCpf_Nde	TGAACATATGATGCAAGAGCGTAAAAAAATATCGCATC
Rev_RbCpf_Xho	ATCTACTCGAGATTATTCGCCATATCATTCTCCTGAACA
Fwd_NLS_Nhe_Hind	CTAGATGCCGAAGAAAAAGCGCAAGGTCA
Rev_NLS_Nhe_Hind	AGCTTGACCTTGCGCTTTTTCTTCGGCAT
Fwd_NLS_Hind	AGCTCCCGAAGAAAAAGCGCAAGGTCA
Rev_NLS_Hind	AGCTTGACCTTGCGCTTTTTCTTCGGG
Fwd_dnmt1_spacer1	TAGAAAGGAAGTCTTGGCTGGCCTTCC
Rev_dnmt1_spacer1	AAAAGGAAGGCCAGCCAAGACTTCCTT
Fwd_dnmt1_spacer2	TAGATCACGGGACTTCTGGCTGAGGTCA
Rev_dnmt1_spacer2	AAAATGACCTCAGCCAGAAGTCCCGTGA
Fwd_dnmt1_spacer3	TAGATCTGATGGTCCATGTCTGTTACTC
Rev_dnmt1_spacer3	AAAAGAGTAACAGACATGGACCATCAGA
Fwd_vegfa_spacer	TAGATCTAGGAATATTGAAGGGGGCAGG
Rev_vegfa_spacer	AAAACCTGCCCCCTTCAATATTCCTAGA
Fwd_emx1_spacer	TAGATTCCTCCGGTTCTGGAACCACACC
Rev_emx1_spacer	AAAAGGTGTGGTTCCAGAACCGGAGGAA

Fwd_dnmt1_del_ngs	GACAGACACACGGAGTGTCTAGCT
Rev_dnmt1_del_ngs	AATTTGGCTCAGCAGGCACC
Fwd_dnmt1_sp1_ngs	CAAGGGCAGCTCAGTGGTGACTT
Rev_dnmt1_sp1_ngs	CTGAGTCACGTGAGTTGATCCCCAT
Fwd_dnmt1_sp2_ngs	GACAGACACACGGAGTGTCTAGCT
Rev_dnmt1_sp2_ngs	GAAGCTGTTGTGTGAGGTTCGCTTATC
Fwd_dnmt1_sp3_ngs	CAGAACTAGTCCTTAGCAGCT
Rev_dnmt1_sp3_ngs	CTCTGGGGACCGTTTGAG
Fwd_vegfa_ngs	GGGTCACTCCAGGATTCC
Rev_vegfa_ngs	CCAAGGTTCACAGCCTGAAA
Fwd_emx1_ngs	GCCTCCTGAGTTTCTCATCTG
Rev_emx1_ngs	CTAGTCATTGGAGGTGACATCG
Fwd_SDM_T	TTTGGAATTTTGTGCCACTTCTG
Fwd_SDM_A	TTTGGAATTTAGTGCCACTTCTG
Fwd_SDM_C	TTTGGAATTTCGTGCCACTTCTG
Rev_SDM	GGTTTACCTTGACCCCTATAG
Fwd_PAM_library	GTAAAACGACGGCCAGTGCCGCAGTACTGATCATNNNNNNNNCCCCTCTATTGATCCCCACCTCCAAATATCTCATCAACAACGTCATAGCTGTTTCCTG

Rev_PAM_library	CAGGAAACAGCTATGACGTTGTTGATGAGATATTTGGAGGTGGGGATCAATAGAGGGGNNNNNNNNATGATCAGTACTGCGGCACTGGCCGTCGTTTTAC
Fwd_RbCpf_RT	AGTGACGACTTGAAGGCTGA
Rev_RbCpf_RT	CGTCACATGGCAGCTTAGAC
Fwd_T7_guide	TAATACGACTCACTATAGG
Rev_TCA_guide	CTAAGAAACCATTATTATCAATCTACAAGAGTAGAAATTACCCTATAGTGAGTCGTATTA
Rev_TCG_guide	GGGTTCCGCGCACATTTCCCATCTACAAGAGTAGAAATTACCCTATAGTGAGTCGTATTA
Rev_TCC_guide	CGTCAGGTGGCACTTTTCGGATCTACAAGAGTAGAAATTACCCTATAGTGAGTCGTATTA
Rev_TCT_guide	AAAGTGCCACCTGACGTCTAATCTACAAGAGTAGAAATTACCCTATAGTGAGTCGTATTA
Rev_CTA_guide	TATTTAGAAAAATAAACAAAATCTACAAGAGTAGAAATTACCCTATAGTGAGTCGTATTA
Rev_CTG_guide	AATAATGGTTTCTTAGACGTATCTACAAGAGTAGAAATTACCCTATAGTGAGTCGTATTA
Rev_CTC_guide	CCGTCATCACCGAAACGCGCATCTACAAGAGTAGAAATTACCCTATAGTGAGTCGTATTA
Rev_CTT_guide	AAAAGTGCCACCTGACGTCTATCTACAAGAGTAGAAATTACCCTATAGTGAGTCGTATTA
Rev_CCA_guide	ACATTTCCCCGAAAAGTGCCATCTACAAGAGTAGAAATTACCCTATAGTGAGTCGTATTA
Rev_CCG_guide	AGACGTCAGGTGGCACTTTTATCTACAAGAGTAGAAATTACCCTATAGTGAGTCGTATTA
Rev_CCC_guide	GACGTCAGGTGGCACTTTTCATCTACAAGAGTAGAAATTACCCTATAGTGAGTCGTATTA
Rev_CCT_guide	ATAATGGTTTCTTAGACGTCATCTACAAGAGTAGAAATTACCCTATAGTGAGTCGTATTA
Rev_TTC_guide	GTCAGGTGGCACTTTTCGGGATCTACAAGAGTAGAAATTACCCTATAGTGAGTCGTATTA
crRNA_site1	rUrArArUrUrUrCrUrArCrUrArUrUrGrUrArGrArUrCrCrCrCrUrCrUrArUrUrGrArUrCrCrCrCrArCrC
Fwd_gDNA_site1	TCTTGCACTCATGAGCTGTC
Rev_gDNA_site1	GTTGAGGGTTATGAGAGTAGC

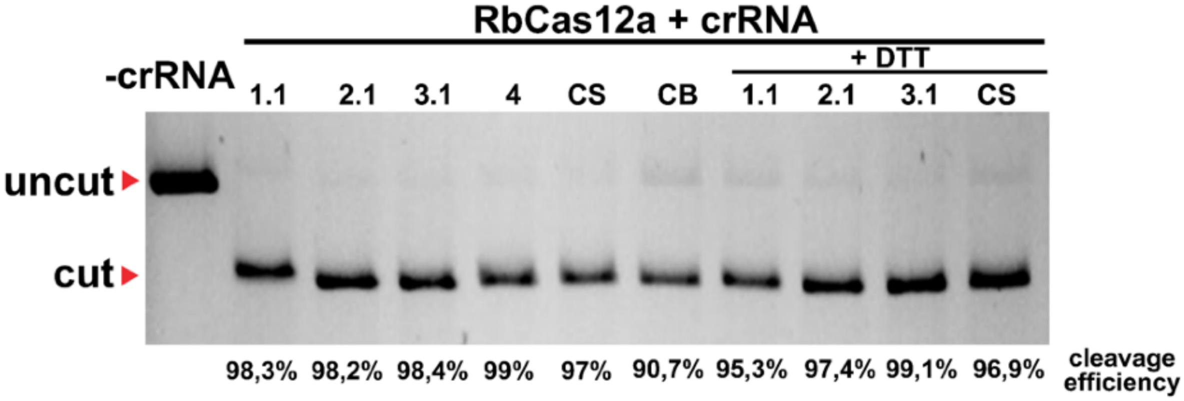

Figure S1. Effect of buffers on RbCas12a crRNA cleavage activity. Target DNA cleavage by RbCas12a programmed with 500 nM crRNA at a 1:3:30 target:RbCas12a:RNA ratio in various conditions. The effect of NEBuffer 1.1 (1.1), NEBuffer 2.1 (2.1), NEBuffer 3.1 (3.1), NEBuffer 4 (4), CutSmart (CS) and cleavage buffer (CB) either supplemented or not with DTT were tested. Mean cleavage efficiencies from three independent experiments are shown below the gel.

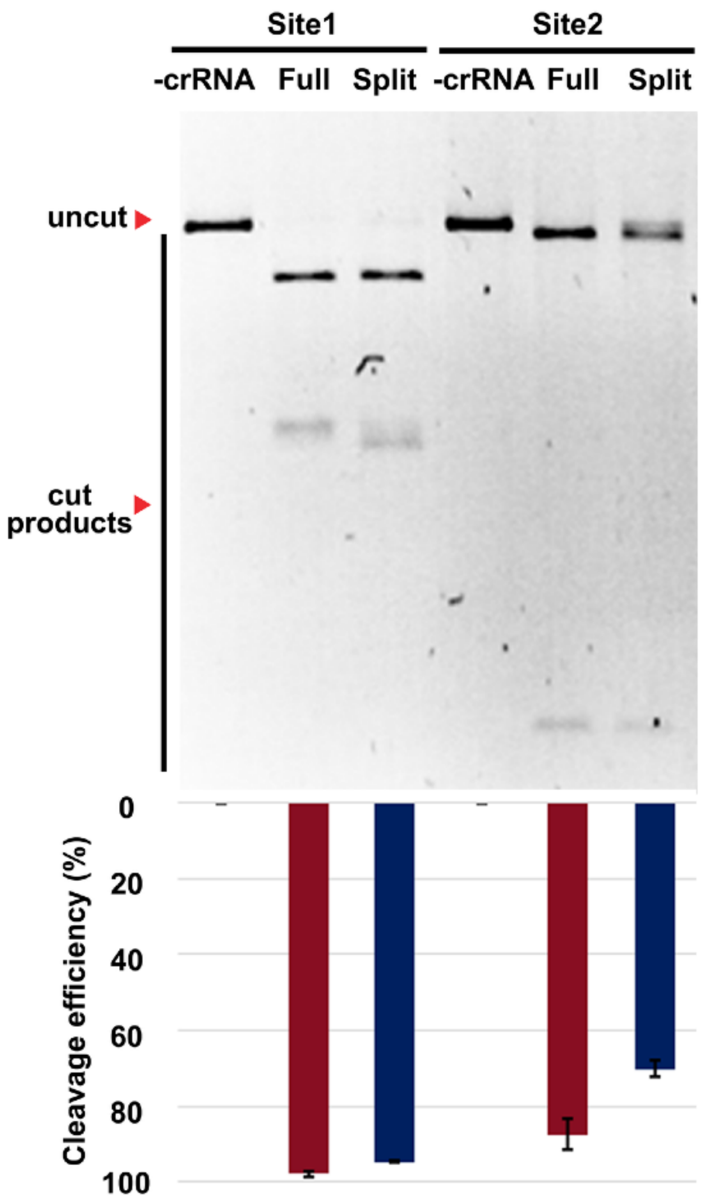

Figure S2. Split crRNA activity is not dependent on the sequence of the crRNA spacer moiety. Target DNA *in vitro* cleavage by RbCas12a loaded with full-sized or split crRNAs (500 nM) bearing different guide sequences ('Site1' and 'Site2' as indicated above the panel). The cleavage efficiencies calculated from one experiment are shown on the bottom. Mean cleavage efficiencies and standard deviations calculated from three independent experiments are shown.

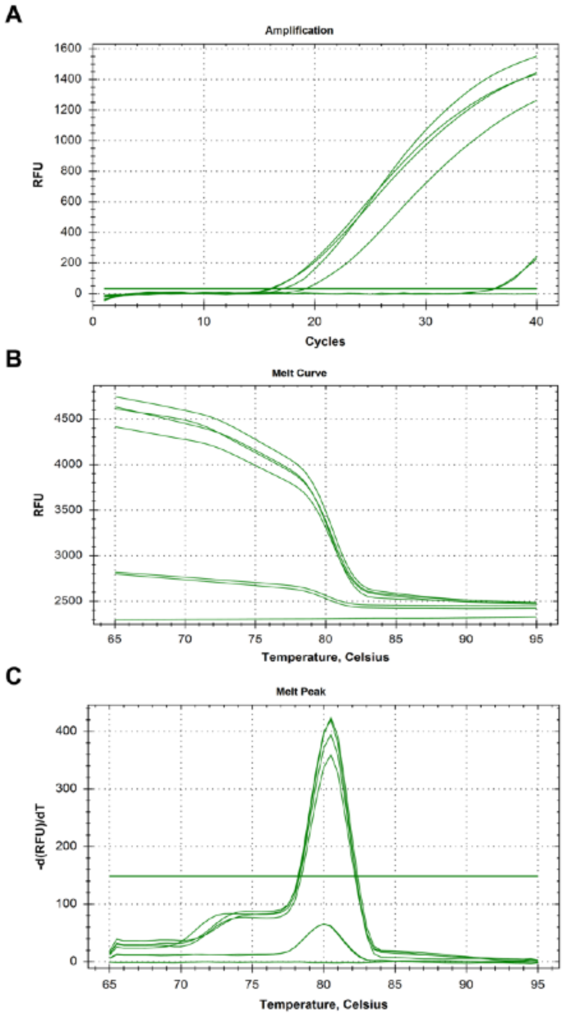

Figure S3. Real-time RT-PCR of RbCas12a mRNA extracted from HEK293T in 24 and 48 hours after transfection (500 ng and 2500 ng of hRbCas12a plasmid vector). **(A)** The signal grows exponentially from cycle 15 in four samples with hRbCas12a plasmid vector. In two mock samples where the plasmid vector was not added the signal is observed from cycle 35. **(B), (C)** Melt curves and peaks indicate specificity of the primer pair.

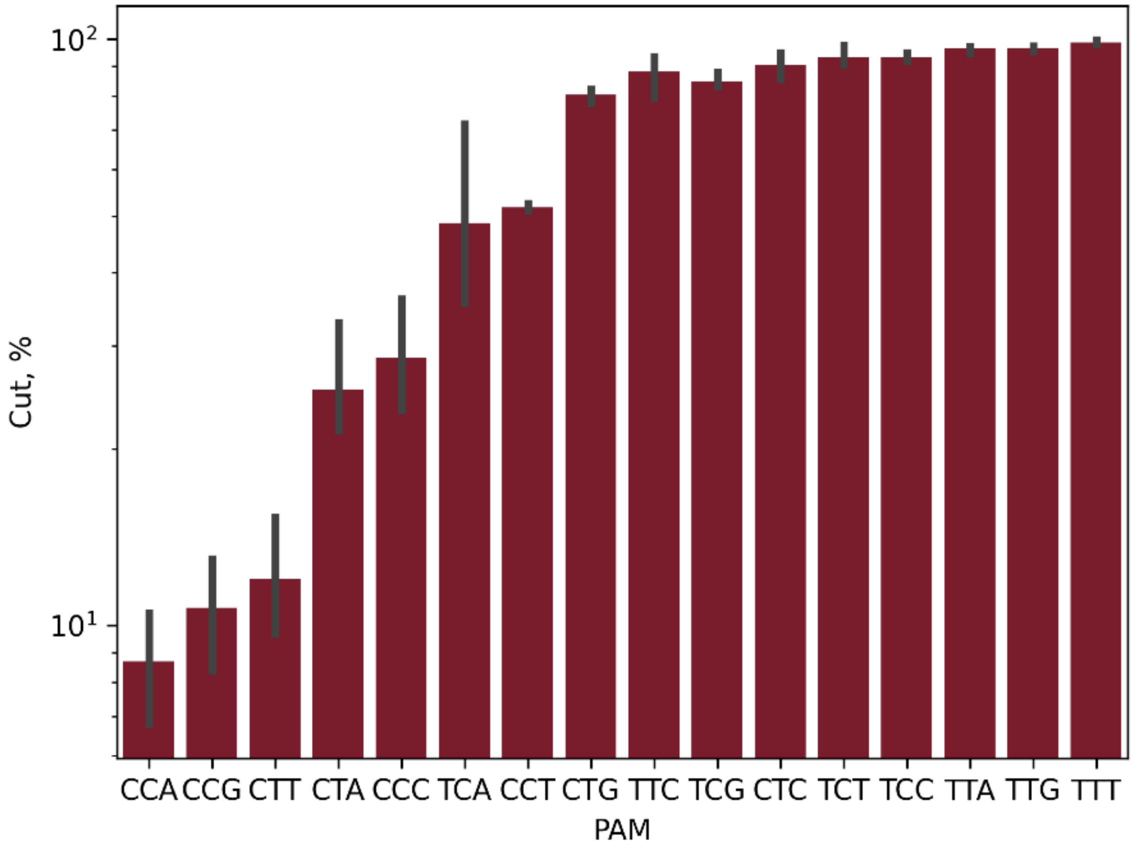

Figure S4. Investigation of RbCas12a 5'-YYN PAM sequence by *in vitro* cleavage assay. Bar plots display the LFC depletion of PAM containing DNA sequences following treatment with RbCas12a.

Nucleotide sequence of RbCas12a

ATGCAAGAGCGTAAAAAAATATCGCATCTTACACACAGAAATTCAGTTAAAAAAACAATTAGGATGCAACTGAATCCTGTAGGT
AAAACAATGGATTATTTCAAGCAAAGCAAATTCTTGAAAATGATGAAAAGCTTAAAGAGAACTATCAGAAAATCAAGGAAAT
AGCAGACAGGTTTTACAGAAATTTAAATGAGGATGTACTTTCAAAAACCGGGTTAGATAAATTAAAAGATTATGCTGAAATTTA
CTATCACTGTAATACGGATGCAGACCGAAAAAGACTTGATGAATGTGCATCGGAATTAAGGAAAGAAATCGTCAAAAATTTTA
AGAATAGAGATGAGTATAACAAACTATTCGATAAAAGGATGATTGAGATAGTTCTTCCCCAGCATCTTAAAAACGAGGACGAAA
AGGAAGTTGTAGCCTCATTTAAAAATTTCACAACATACTTTACAGGTTTCTTCACTAACAGAAAAAATATGTATTCGGACGGAG
AAGAATCCACGGCAATCGCATATAGATGCATTAACGAAAATTTGCCTAAACATCTTGACAATGTCAAGGCCTTTGAAAAAGCAA
TTTCTAAACTATCCAAAAACGCAATTGATGATTTAGATGCCACTTATTCCGGCTTATGCGGTACAAATTTGTATGATGTTTTTACA
GTTGATTATTTTAACTTTTTGCTTCCACAATCCGGAATTACCGAATATAACAAAATCATCGGCGGTTACACAACAAGCGACGGTA
CAAAAGTTAAGGGTATTAACGAATATATAAATTTGTACAATCAACAAGTATCCAAACGGGATAAAATTCCTAATCTTCAAATTTT
GTATAAACAAATTTTAAGTGAGAGTGAAAAGGTATCATTCATACCGCCAAAGTTTGAAGATGACAACGAACTTTTATCGGCTGT
TTCAGAGTTTTACGCAAACGACGAAACCTTTGACGGGATGCCATTAAAAAAAGCAATTGATGAAACAAAGCTATTATTCGGCA
ATTTAGATAATTCCTCTCTTAATGGAATTTACATTCAAAATGACCGATCCGTGACAAATCTGTCAAACAGTATGTTCGGTTCTTGG
TCGGTAATAGAAGATTTATGGAACAAAAATTATGACTCCGTTAATTCAAACAGCAGAATCAAAGATATTCAAAAGCGTGAAGAC
AAAAGAAAAAAAGCATACAAAGCAGAAAAGAAACTTTCACTTTCATTTTTACAGGTTTTGATTTCCAATTCCGAAAATGATGA
AATCAGAAAAAAGTCTATCGTAGATTACTACAAGACTTCTTTAATGCAACTTACCAACAATTTATCAGACAAATACAACGAAGC
AGCACCTCTGTTCAGTGAAAATTACGATAATGAAAAAGGTTTGAAAAATGACGATAAATCTATTTCATTAATTAAAAATTTTCTT
GATGCCATAAAAGAAATTGAAAAATTCATAAAGCCTTTGTCCGAAACTAATATTACAGGTGAGAAAAATGATTTGTTTTACAGT
CAGTTCACACCATTACTTGATAATATCAGCAGAATAGACATATTATATGATAAGGTCAGAAACTATGTTACACAAAAACCGTTTT
CAACCGATAAAATCAAGCTTAACTTTGACAATTACCAGCTATTAAACGGCTGGGATAAAGACAAAGAAAGAGAGTACGGAGCC
GTTTTGCTTTGTAAAGATGAAAAGTATTATCTTGCAATCATAGATAAAAGCAATAATCGTATTTTGGAAAATATTGATTTTCAAGA
CTGCGATGAAAGCGATTGTTACGAAAAGATAATTTACAAGCTTCTCCCCACTCCAAATAAAATGCTTCCAAAAGTTTTCTTTGC
AAAAAAGCACAAAAAAACTTTTGTCACCGTCAGACGAAATACTTAAAATTTATAAAAGCGGCACTTTCAAAAAAGGTGATAAGT
TCAGCCTTGATGATTGCCATAAGTTAATTGATTTCTACAAAGAATCATTCAAAAAGTACCCAAAATGGTTAATTTATAACTTTAAA
TTCAAAAAAACAAACGAATATAACGATATCCGCGAATTTTATAATGATGTTGCTTTACAGGGATATAATATTTCAAAAATGAAAA
TCCCGACATCATTTATTGACAAACTTGTAGATGAAGGAAAAATCTATCTTTTCCAACTCTACAACAAAGACTTTTCACCGCATAG
CAAGGGTACTCCTAATCTGCATACACTTTATTTTAAAATGTTATTTGATGAAAGAAATCTTGAAGATGTGGTGTATAGGCTTAAC
GGTGAGGCAGAAATGTTTTATCGTCCTGCAAGTATAAAATATGACAAACCCACTCATCCCAAAAACACACCGATAAAAAATAA
AAATACACTCAATGATAAAAAAGCAAGCACTTTTCCTTATGACTTAATTAAAGATAAACGCTACACTAAATGGCAGTTTTCACTT
CACTTCCCTATTACCATGAATTTTAAAGCTCCGGATAGGGCAATGATCAATGATGATGTCAGAAATCTGCTGAAATCCTGCAACA
ACAATTTCATCATAGGAATTGACAGAGGCGAAAGAAACTTGCTTTATGTCAGCGTAATTGACAGCAACGGTGCTATAATATATC
AGCACTCACTCAATATTATCGGAAACAAGTTTAAAGGAAAAACATACGAAACTAACTACCAGGAAAAACTTGCAACAAGAGA
AAAAGAGCGTACGGAACAGCGCCGTAACTGGAAAGCAATTGAGAGTATAAAAGAACTCAAAGAGGGCTATATCAGTCAGGCT
GTGCATGTTATATGTCAGCTTGTTGTCAAGTACGATGCAATCATCGTTATGGAAAAGCTGACTGACGGATTCAAACGAGGCAGA
ACAAAGTTTGAAAAACAGGTTTATCAGAAATTTGAAAAAATGCTGATTGACAAACTTAATTACTATGTTGACAAAAAGCTTGAT
CCCGATGAAGAAGGCGGTTTACTTCATGCCTACCAGCTTACGAACAAGCTTGAGAGCTTTGATAAGCTTGGTACGCAAAGCGG
TTTTATTTTCTATGTTCGTCCTGATTTTACAAGCAAAATTGATCCCGTTACCGGCTTTGTAAATTTGTTGTACCCTCGATATGAAA
ACATTGACAAAGCCAAAGATATGATTTCAAGATTTGACGATATAAGATACAATGCCGGCGAGGACTTTTTTGAATTTGACATTGA
TTACGATAAGTTTCCAAAGACTGCGTCTGACTATCGCAAAAAGTGGACAATCTGTACTAACGGCGAAAGGATTGAAGCTTTCA
GAAATCCCGCAAACAATAACGAATGGAGTTATCGTACAATAATTCTTGCAGAAAAATTCAAAGAATTATTTGATAACAATTCTAT
AAATTATCGTGATTCTGACGATTTGAAAGCTGAAATTCTTTCACAGACAAAGGGCAAATTTTTTGAGGATTTCTTCAAATTATTA
AGACTTACCCTACAGATGCGAAACAGTAACCCTGAAACAGGCGAGGACCGTATTCTTTCTCCCGTCAAGGACAAAAACGGCA
ATTTTTACGACAGTTCAAAATATGATGAAAAGAGCAAGCTTCCGTGTGACGCCGATGCAAACGGTGCGTACAACATTGCCCGC
AAAGGTTTGTGGATTGTTGAACAATTCAAAAAATCCGATAATGTTTCAACTGTCGAACCGGTAATTCACAATGACAAATGGCTG
AAATTTGTTCAGGAGAATGATATGGCGAATAATCTCGAG

Amino-acid sequence of RbCas12a

MQERKKISHLTHRNSVKKTIRMQLNPVGKTMDYFQAKQILENDEKLKENYQKIKEIADRFYRNLNEDVLSKTGLDKLKDYAEIYY
HCNTDADRKRLDECASELRKEIVKNFKNRDEYNKLFDKRMIEIVLPQHLKNEDEKEVVASFKNFTTYFTGFFTNRKNMYSDGEEST
AIAYRCINENLPKHLDNVKAFEKAISKLSKNAIDDLDATYSGLCGTNLYDVFTVDYFNFLLPQSGITEYNKIIGGYTTSDGTKVKGIN
EYINLYNQQVSKRDKIPNLQILYKQILSESEKVSFIPPKFEDDNELLSAVSEFYANDETFDGMPLKKAIDETKLLFGNLDNSSLNGIYI
QNDRSVTNLSNSMFGSWSVIEDLWNKNYDSVNSNSRIKDIQKREDKRKKAYKAEKKLSLSFLQVLISNSENDEIRKKSIVDYYKTS
LMQLTNNLSDKYNEAAPLFSENYDNEKGLKNDDKSISLIKNFLDAIKEIEKFIKPLSETNITGEKNDLFYSQFTPLLDNISRIDILYDKV
RNYVTQKPFSTDKIKLNFDNYQLLNGWDKDKEREYGAVLLCKDEKYYLAIIDKSNNRILENIDFQDCDESDCYEKIIYKLLPTPNK
MLPKVFFAKKHKKLLSPSDEILKIYKSGTFKKGDKFSLDDCHKLIDFYKESFKKYPKWLIYNFKFKKTNEYNDIREFYNDVALQGY
NISKMKIPTSFIDKLVDEGKIYLFQLYNKDFSPHSKGTPNLHTLYFKMLFDERNLEDVVYRLNGEAEMFYRPASIKYDKPTHPKNTPI
KNKNTLNDKKASTFPYDLIKDKRYTKWQFSLHFPITMNFKAPDRAMINDDVRNLLKSCNNNFIIGIDRGERNLLYVSVIDSNGAIIY
QHSLNIIGNKFKGKTYETNYQEKLATREKERTEQRRNWKAIESIKELKEGYISQAVHVICQLVVKYDAIIVMEKLTDGFKRGRTKFE
KQVYQKFEKMLIDKLNYYVDKKLDPDEEGGLLHAYQLTNKLESFDKLGTQSGFIFYVRPDFTSKIDPVTGFVNLLYPRYENIDKAK
DMISRFDDIRYNAGEDFFEFDIDYDKFPKTASDYRKKWTICTNGERIEAFRNPANNNEWSYRTIILAEKFKELFDNNSINYRDSDDLK
AEILSQTKGKFFEDFFKLLRLTLQMRNSNPETGEDRILSPVKDKNGNFYDSSKYDEKSKLPCDADANGAYNIARKGLWIVEQFKKS
DNVSTVEPVIHNDKWLKFVQENDMANNLE

Human optimized nucleotide sequence of RbCas12a with nuclear localization signals

(SV40 NLS is highlighted red, nucleoplasmin NLS is blue)

ATGCCGAAGAAAAAGCGCAAGGTCAAGCTCCCGAAGAAAAAGCGCAAGGTCAAGCTCCCGAAGAAAAAGCGCAAGGTCAA
GCTTATGCAAGAACGCAAGAAGATTAGTCATCTGACCCATAGAAACTCCGTGAAGAAGACCATCCGTATGCAATTAAACCCGG
TCGGCAAGACCATGGACTACTTCCAAGCCAAGCAAATCCTGGAGAACGACGAGAAGCTGAAGGAAAACTACCAAAAGATCA
AGGAGATTGCCGACCGTTTCTATAGAAACTTAAACGAAGACGTCCTGAGCAAGACTGGTTTGGACAAGTTAAAGGACTACGCT
GAGATCTATTACCATTGTAACACGGACGCCGACCGTAAGAGACTGGACGAGTGTGCCAGTGAGTTACGTAAGGAGATCGTAAA
GAACTTCAAGAACAGAGACGAATACAACAAGCTATTTGACAAGCGTATGATCGAAATTGTGCTGCCGCAACATCTGAAGAACG
AAGACGAGAAGGAGGTGGTCGCGTCTTTCAAGAACTTTACCACCTACTTCACCGGCTTTTTTACTAACAGAAAGAACATGTAC
AGTGACGGAGAGGAGTCGACGGCCATCGCCTACAGATGTATCAACGAGAACTTACCGAAGCATCTGGACAACGTAAAGGCAT
TCGAGAAGGCTATCAGTAAGCTATCGAAGAACGCCATCGACGACTTAGACGCAACTTACTCGGGCTTATGTGGAACCAACTTG
TACGACGTGTTCACCGTGGACTACTTCAACTTCTTGCTGCCGCAATCGGGAATCACTGAGTACAACAAGATCATCGGCGGCTAC
ACCACCTCTGACGGCACCAAGGTGAAAGGTATCAACGAGTACATTAACTTGTACAACCAACAAGTCTCGAAGCGTGACAAGA
TCCCGAACCTGCAAATCTTGTACAAGCAAATCTTAAGTGAAAGTGAGAAGGTCAGCTTTATTCCACCGAAGTTCGAGGACGAC
AACGAGCTGTTAAGTGCTGTGAGCGAATTTTACGCCAACGACGAGACTTTCGACGGTATGCCGTTAAAGAAGGCCATCGACGA
GACCAAGCTATTATTTGGCAACTTAGACAACTCGAGTCTGAACGGAATCTACATCCAAAACGACCGTTCGGTTACCAACTTAAG
CAACAGTATGTTTGGCAGTTGGAGTGTGATTGAGGACTTATGGAACAAGAACTACGACTCGGTGAACTCTAACTCTAGAATCA
AGGACATCCAAAAGCGCGAGGACAAGAGAAAGAAGGCCTACAAGGCCGAGAAGAAGCTGAGCCTGAGCTTCTTACAAGTGT
TGATCTCGAACTCGGAGAACGACGAGATCAGAAAGAAGAGTATCGTCGACTACTACAAGACTAGTTTAATGCAACTGACTAAC
AACTTAAGCGACAAGTACAACGAGGCCGCCCCGTTATTTAGTGAGAACTACGACAACGAGAAGGGCTTGAAGAACGACGACA
AGAGTATCAGCTTAATCAAGAACTTCCTGGACGCAATTAAGGAGATCGAGAAGTTTATTAAGCCGTTGTCGGAGACTAACATCA
CCGGCGAAAAGAACGACTTGTTCTACAGTCAATTTACCCCGTTACTGGACAACATCTCTAGAATTGACATTTTATACGACAAGG
TAAGAAACTACGTGACCCAAAAGCCATTCAGCACTGACAAGATCAAGCTGAACTTCGACAACTACCAACTATTAAACGGCTGG
GACAAGGACAAGGAGAGAGAATACGGAGCGGTGTTGCTGTGTAAGGACGAGAAGTACTACCTGGCCATCATTGACAAGTCTA
ACAACCGCATCTTGGAGAACATCGACTTCCAAGACTGTGACGAGTCTGACTGTTACGAGAAGATTATCTACAAGCTGCTTCCG
ACTCCGAACAAGATGCTGCCGAAGGTGTTTTTCGCCAAGAAGCATAAGAAGCTGTTGAGCCCAAGCGACGAGATTCTGAAGA
TCTACAAGTCTGGCACTTTTAAGAAGGGCGACAAGTTTTCTCTGGACGACTGTCATAAGTTAATCGACTTTTACAAGGAGAGCT
TTAAGAAGTACCCGAAGTGGTTAATCTACAACTTCAAGTTTAAGAAGACCAACGAGTACAACGACATCCGTGAGTTCTACAAC
GACGTGGCTTTACAAGGTTACAACATCAGCAAGATGAAGATCCCGACCTCTTTCATCGACAAGCTGGTCGACGAGGGAAAGAT
CTACCTGTTTCAACTTTACAACAAGGACTTCAGCCCACATTCTAAGGGCACTCCGAACTTACATACCCTGTACTTCAAGATGTTA
TTCGACGAGAGAAACCTGGAGGACGTTGTTTACCGTCTGAACGGCGAAGCCGAGATGTTCTACCGCCCGGCCAGTATTAAGTA
CGACAAGCCGACTCATCCGAAGAACACCCCAATTAAGAACAAGAACACCCTTAACGACAAGAAGGCCTCTACTTTCCCGTAC
GACTTAATCAAGGACAAGCGTTACACTAAGTGGCAATTCAGCCTGCATTTTCCGATCACTATGAACTTCAAGGCTCCAGACCGT
GCCATGATTAACGACGACGTAAGAAACTTATTAAAGTCGTGTAACAACAACTTTATCATTGGAATCGACAGAGGCGAGAGAAA
CTTGCTGTACGTATCTGTCATCGACTCTAACGGCGCTATTATTTACCAACATAGCCTTAACATCATCGGAAACAAGTTCAAGGGA
AAGACCTACGAGACTAACTACCAAGAGAAGCTGGCCACCAGAGAGAAGGAACGCACGGAGCAACGTCGCAACTGGAAGGCC
ATCGAAAGTATTAAGGAGCTTAAGGAAGGCTACATCAGTCAAGCTGTTCATGTGATTTGTCAACTGGTGGTAAAGTACGACGC
CATCATCGTGATGGAGAAGTTAACTGACGGATTTAAGCGTGGCAGAACCAAGTTCGAGAAGCAAGTGTACCAAAAGTTCGAG
AAGATGTTAATCGACAAGCTGAACTACTACGTGGACAAGAAGCTGGACCCGGACGAGGAGGGCGGCTTACTGCATGCATACC
AACTGACGAACAAGCTGGAATCTTTCGACAAGCTGGGCACGCAATCTGGCTTCATCTTTTACGTGCGCCCGGACTTCACCTCT
AAGATCGACCCGGTGACTGGCTTCGTCAACTTGTTGTACCCGCGTTACGAGAACATCGACAAGGCAAAGGACATGATCAGCA
GATTCGACGACATTAGATACAACGCAGGCGAAGACTTCTTCGAGTTCGACATCGACTACGACAAGTTCCCGAAGACTGCGAGT
GACTACCGTAAGAAGTGGACCATCTGTACTAACGGCGAGCGTATCGAGGCTTTTAGAAACCCGGCCAACAACAACGAGTGGA
GTTACCGCACCATTATCCTGGCCGAGAAGTTTAAGGAGTTATTCGACAACAACAGTATTAACTACCGCGACAGTGACGACTTGA
AGGCTGAGATCCTGAGCCAAACCAAGGGCAAGTTCTTCGAAGACTTTTTTAAGTTATTAAGACTGACTCTACAAATGCGTAAC
AGTAACCCGGAGACCGGCGAAGACCGCATCCTGAGTCCGGTAAAGGACAAGAACGGAAACTTCTACGACAGTAGCAAGTAC
GACGAGAAGTCTAAGCTGCCATGTGACGCAGACGCCAACGGCGCGTACAACATCGCACGTAAGGGCTTGTGGATCGTGGAGC
AATTTAAGAAGTCGGACAACGTGAGCACTGTAGAGCCAGTCATCCATAACGACAAGTGGTTAAAGTTCGTGCAAGAAAACGA
CATGGCGAACAACAAGCGGCCTGCGGCTACTAAGAAGGCTGGGCAGGCTAAGAAGAAGAAG
